# Lin28 Mediates Paclitaxel Resistance by Modulating p21, Rb and Let-7a miRNA in Breast Cancer Cells

**DOI:** 10.1371/journal.pone.0040008

**Published:** 2012-07-09

**Authors:** Kezhen Lv, Liqun Liu, Linbo Wang, Jiren Yu, Xiaojiao Liu, Yongxia Cheng, Minjun Dong, Rongyue Teng, Linjiao Wu, Peifen Fu, Wuguo Deng, Wenxian Hu, Lisong Teng

**Affiliations:** 1 Department of Gastrointestinal Surgery, The First Affiliated Hospital, College of Medicine, Zhejiang University, Hangzhou, China; 2 Key Laboratory of Biotherapy of Zhejiang Province, Department of Surgical Oncology, Sir Run Run Shaw Hospital, College of Medicine, Zhejiang University, Hangzhou, China; 3 Department of General Surgery, The First Affiliated Hospital-Huangpu Hospital, Sun Yat-Sen University, Guangzhou, China; 4 Institute of Cancer Stem Cell, Dalian Medical University Cancer Center, Dalian, China; 5 State Key Laboratory of Oncology in South China, Sun Yat-Sen University Cancer Center, Guangzhou, China; King Faisal Specialist Hospital & Research Center, Saudi Arabia

## Abstract

Resistance to chemotherapy is a major obstacle for the effective treatment of cancers. Lin28 has been shown to contribute to tumor relapse after chemotherapy; however, the relationship between Lin28 and chemoresistance remained unknown. In this study, we investigated the association of Lin28 with paclitaxel resistance and identified the underlying mechanisms of action of Lin28 in human breast cancer cell lines and tumor tissues. We found that the expression level of Lin28 was closely associated with the resistance to paclitaxel treatment. The T47D cancer cell line, which highly expresses Lin28, is more resistant to paclitaxel than the MCF7, Bcap-37 or SK-BR-3 cancer cell lines, which had low-level expression of Lin28. Knocking down of Lin28 in Lin28 high expression T47D cells increased the sensitivity to paclitaxel treatment, while stable expression of Lin28 in breast cancer cells effectively attenuated the sensitivity to paclitaxel treatment, resulting in a significant increase of IC50 values of paclitaxel. Transfection with Lin28 also significantly inhibited paclitaxel-induced apoptosis. We also found that Lin28 expression was dramatically increased in tumor tissues after neoadjuvant chemotherapy or in local relapse or metastatic breast cancer tissues. Moreover, further studies showed that p21, Rb and Let-7 miRNA were the molecular targets of Lin28. Overexpression of Lin28 in breast cancer cells considerably induced p21 and Rb expression and inhibited Let-7 miRNA levels. Our results indicate that Lin28 expression might be one mechanism underlying paclitaxel resistance in breast cancer, and Lin28 could be a potential target for overcoming paclitaxel resistance in breast cancer.

## Introduction

Resistance to chemotherapy is a major obstacle for the effective treatment of cancers. Although many anticancer therapies can alter tumor growth, in most cases the effect is not long-lasting. Approximately 30% of the women diagnosed with early-stage disease eventually progress to metastatic breast cancer, for which therapeutic options are limited. Current recommendations for first-line chemotherapy include treatment with taxanes such as paclitaxel or docetaxel. These regimens typically give response rates of 30 to 70%, but the responses are often not permanent, with a time to progression of 6 to 10 months [1]; [2]. Thus, there is an urgent need to explore taxane resistance mechanisms to improve response rates and potentially extend survival.

Lin28 is a marker of cancer stem cells, which contribute to tumor relapse after traditional treatments including chemotherapy [3]. It is highly expressed in some tumors, such as hepatocellular carcinoma [4]. Overexpression of Lin28 has been shown to promote cancer cell proliferation [5]; [6]. However, there is no information available to show a relationship between the dysregulation of Lin28 and the chemoresistance of cancer cells. Moreover, the mechanism of action of Lin28 and its molecular targets are not completely understood.

Herein, we investigated the expression of Lin28 in various breast cancer cell lines and tumor tissues. We postulate that Lin28 expression is implicated in chemoresistance. To investigate the underlying mechanisms, we analyzed the role of Lin28 in the regulation of p21, RB, cyclin B1, Akt and Let-7 miRNA. The present study suggests that Lin28 may be a potential target to overcome chemoresistance and provides a scientific basis for further investigation of mechanisms of chemoresistance.

## Results

### Lin28 Expression is Implicated in Paclitaxel Resistance in Breast Cancer Cell Lines

**Figure 1:**
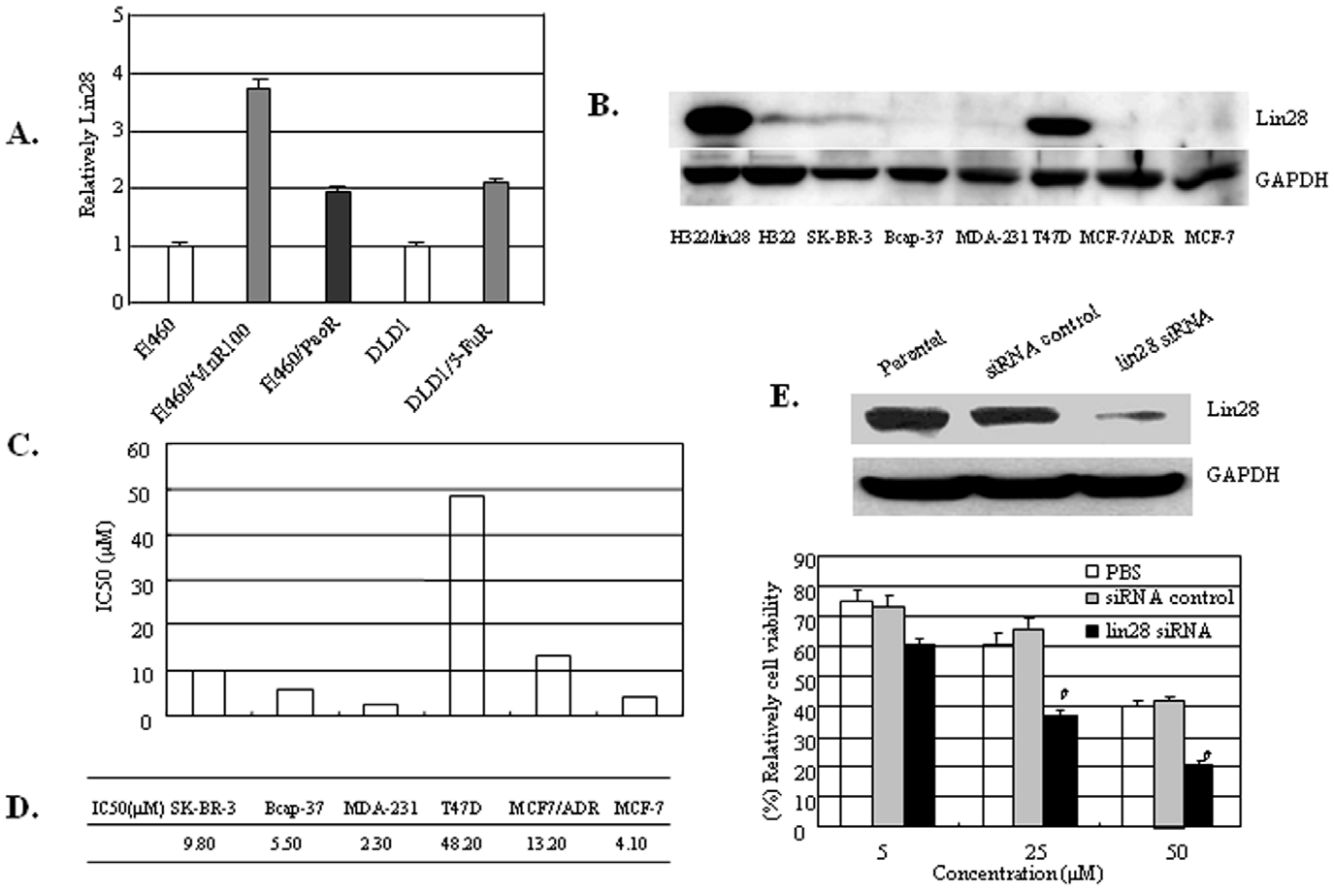
Lin28 expression is associated with sensitivity to paclitaxel in breast cancer cells. (A). Lin28 expression was determined in the indicated cancer cell lines and their chemoresistant subclones by qPCR. (B). Lin28 expression was determined in the indicated breast cancer cell lines by Western blotting. GAPDH expression was used as a loading control, and the stable H322/Lin28 lung cancer cell line was used as positive control for Lin28 expression. (C, D). IC50 values of paclitaxel in the indicated breast cancer cell lines. Cells were treated with paclitaxel for 48 h and cell viability was determined by an MTT assay. Cells treated with PBS were used as a control. (E) Knocking down of lin28 by lin28 siRNA reverses paclitexal resistance in highly lin28 expression T47D cell line. Parental and siRNA control transfected T47D were used as controls. #, P<0.05.

Lin28 is a marker of cancer stem cells, which contribute to tumor relapse after traditional treatment including chemotherapy. To determine whether Lin28 expression is associated with chemoresistance of tumor cells, we examined the expression of Lin28 by RT-PCR in the human lung cancer cell line H460 and its corresponding paclitaxel-resistant (H460/PacR) and vincristine-resistant (H460/VinR) subclones, and in the human colon cancer cell line DLD1 and its corresponding 5-Fu-resistant subclones (DLD1/5-FuR). We found that Lin28 is highly expressed in the chemoresistant lung and colon cancer cell lines compared with the parental cells (Fig. 1A), indicating that Lin28 is implicated in the chemoresistance of tumor cells.

To determine whether Lin28 expression is also associated with chemoresistance in breast cancer, we also measured the expression of Lin28 proteins in six breast cancer cell lines by Western blot and determined their sensitivities to the chemotherapeutic drug paclitaxel, which is widely used in breast cancer treatment. Our results showed that Lin28 was highly expressed in T47D cancer cells, whereas its expression was relatively lower in MCF7, Bcap-37 and SK-BR-3 cancer cells (Fig. 1B). Further studies showed that the protein expression level of Lin28 was closely associated with how resistant the cells were to paclitaxel treatment. The IC50 values of four cell lines to paclitaxel treatment were 48.20 µM for T47D cells, 4.40 µM for MCF-7 cells, 5.50 µM for Bcap-37 cells and 9.80 µM for SK-BR-3 cells (Fig. 1C, 1D).

To further confirm the role of lin28 expression in regulating paclitaxel resistance in breast cancer, we knocked down lin28 expression by lin28 siRNA in T47D cancer cells with lin28 highly expression, and determined their sensitivity to paclitaxel. The IC50 of paclitaxel in lin28 knocking down T47D cells was dramatically downregulated compared to siRNA control transfected and PBS treated cells (*P*<0.05) (Fig. 1E). This result suggests that downregulation of lin28 in breast cancer cells can increase their sensitivity to paclitaxel treatment.

**Figure 2:**
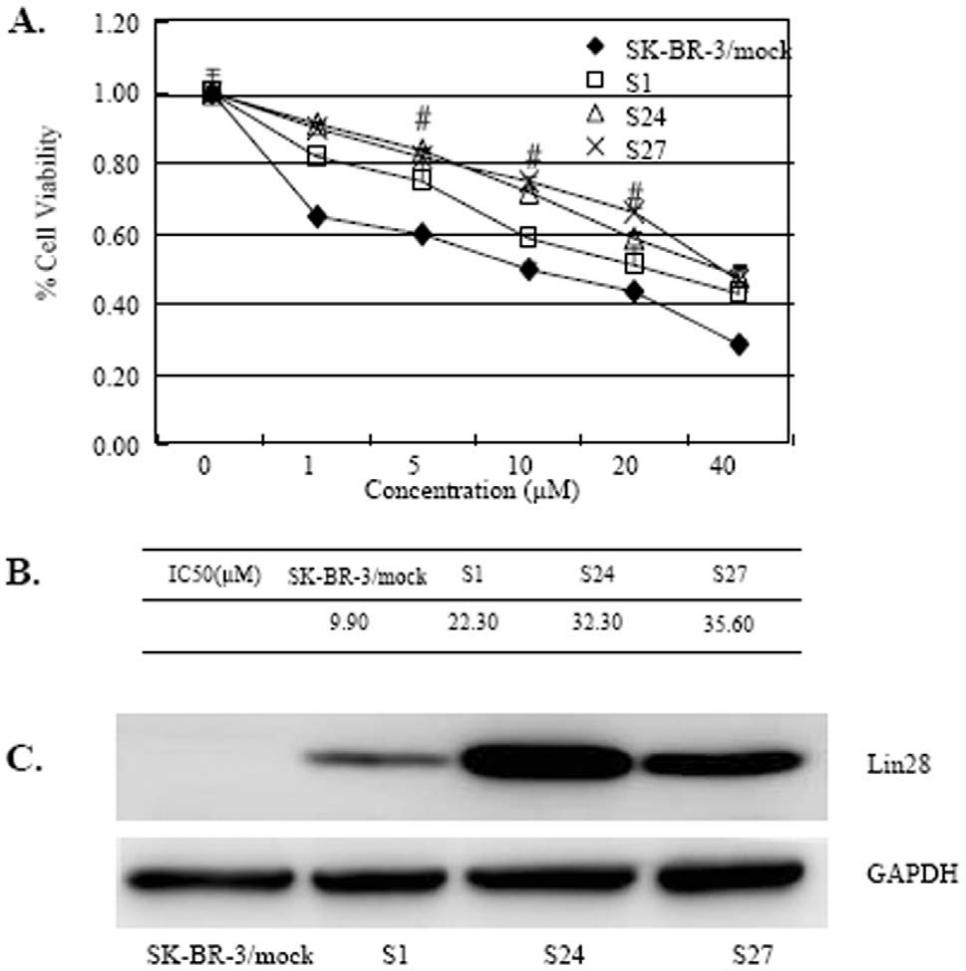
The SK-BR-3 cell line with stable Lin28 expression is relatively tolerant of paclitaxel treatment. (A). Cell viability of SK-BR-3 and its subclones S1, S24 and S27, which stably express Lin28. Cells were treated with paclitaxel for 48 h and cell viability was determined using the MTT assay. SK-BR-3 cells transfected with empty vector (SK-BR-3/mock) were used as a mock control. Each data point represents the mean SD of three independent experiments. Compared to mock controls, #, P<0.05. (B). IC50 values for paclitaxel. (C). Lin28 expression in the indicated cell lines was determined by Western blot. GAPDH expression was used as a loading control.

To further confirm the role of Lin28 expression in regulating paclitaxel resistance in breast cancer, we established three SK-BR-3 clones (S1, S24, S27) and one Bcap-37 clone (B1) and evaluated the sensitivities of these subclones to paclitaxel. The expression of Lin28 was confirmed by Western blotting. We showed that the parental SK-BR-3 cell did not express Lin28, but the three clones (S1, S24, S27) had stable expression of Lin28 (Fig. 2C). We also found that these stable Lin28 expression clones were more resistant to paclitaxel compared to the control cells (Fig. 2A). The IC50 values were 9.90 µM for the mock-transfected SK-BR-3 cells and 22.30 µM–35.60 µM for the three SK-BR-3 clones stably expressing Lin28 (Fig. 2B). Our results also showed that the IC50 values were 6.10 µM for the mock-transfected Bcap-37 cells and 28.30 µM for the Bcap-37 B1 clone (data not shown). Moreover, the results showed that of the three SK-BR-3 clones with stable Lin28 expression, the S1 clone had lower Lin28 expression than the S24 and S27 clones (Fig. 2C). The clones that expressed high levels of Lin28 (S24 and S27) were more resistant than the S1 clone to paclitaxel treatment (Fig. 2B). These results indicate that Lin28 expression was positively correlated with paclitaxel resistance.

Next, we examined the effect of treatment of breast cancer cells with paclitaxel on Lin28 protein expression in T47D. MCF7, Bcap-37 and SK-BR-3 cells and found that the treatment with paclitaxel did not alter the levels of Lin 28 protein expression (data not shown).

### Lin28 Transfection Inhibits Paclitaxel-induced Apoptosis

**Figure 3:**
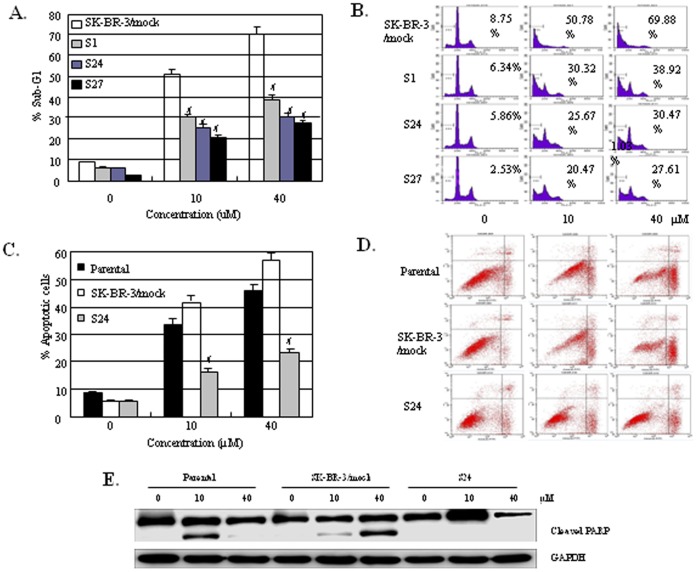
Overexpression of Lin28 decreases the paclitaxel-induced apoptosis in SK-BR-3 cell lines stably expressing Lin28. Cells were treated with paclitaxel at doses ranging from 0 to 40 µM for 48 h, and apoptosis was analyzed. The empty vector-transfected cells were used as controls. (**A**). Sub-G1 peak compared with control in SK-BR-3 lin28 stable expression clones #, *P*<0.01. (**B**). Representative of PI staining examined in SK-BR-3 lin28 stable expression clones. (**C**). Apoptosis compared with control in SK-BR-3 lin28 stable expression S24 clone. #, *P*<0.01. (**D**). Representative of PI-Annexin δ examined in S24 clone. (**E**) PARP expression was determined in indicated cells by Western blotting.

Paclitaxel has been shown to induce apoptosis in breast cancer cells, so we next determined the effect of Lin28 transfection on paclitaxel-induced apoptosis. The three SK-BR-3 clones with stable Lin28 expression exhibited a significantly reduced level of paclitaxel-induced cell death compared to the parental SK-BR-3 cells (*P*<0.01), resulting in a reduction from 50.78% to 20.47∼30.32% and 69.88% to 27.61∼38.92% at the dose of 10 µM and 40 µM paclitaxel, respectively (Fig. 3A, 3B). Similarly, the paclitaxel-mediated cell death in the Bcap37 clone with stable Lin28 expression was significantly inhibited compared to that seen in the parental Bcap37 cells (*P*<0.01), with a decrease of apoptosis from 28.02% to 5.39% and 32.08% to 8.95% at 5 µM and 20 µM paclitaxel, respectively.

To further clarify whether these dead cells were induced by apoptosis, we used PI-Annexin V double staining to measure apoptotic cells and measured apoptotic protein PARP gene expression by Western blotting. We found apoptotic cells were dramatically decreased in lin28 stable expression clone S24 from 42.27% to 17.62% and 58.81% to 24.71% at the dose of 10 µM and 40 µM paclitaxel, respectively (*P*<0.01) (Fig. 3C, 3D). From these results, we can calculate that percentage of apoptotic cells were nearly to 75∼82% among paclitaxel-induced S24 dead cells. Most of breast cancer cell death induced by paclitaxel was contributed to apoptosis. Moreover, we measured cleaved PAPR expression, which means to develop apoptosis, was upregulated after paclitaxel treatment in the parental and mock control cells, while in lin28 stable expression S24 cells, cleaved PARP expression showed no changement (Fig. 3E). These results indicate that lin28-induced resistance to paclitaxel mostly contributed to inhibition of apoptosis in breast cancer cells.

### Lin28 Expression is Associated with Relapse or Metastasis of Breast Cancer

**Figure 4:**
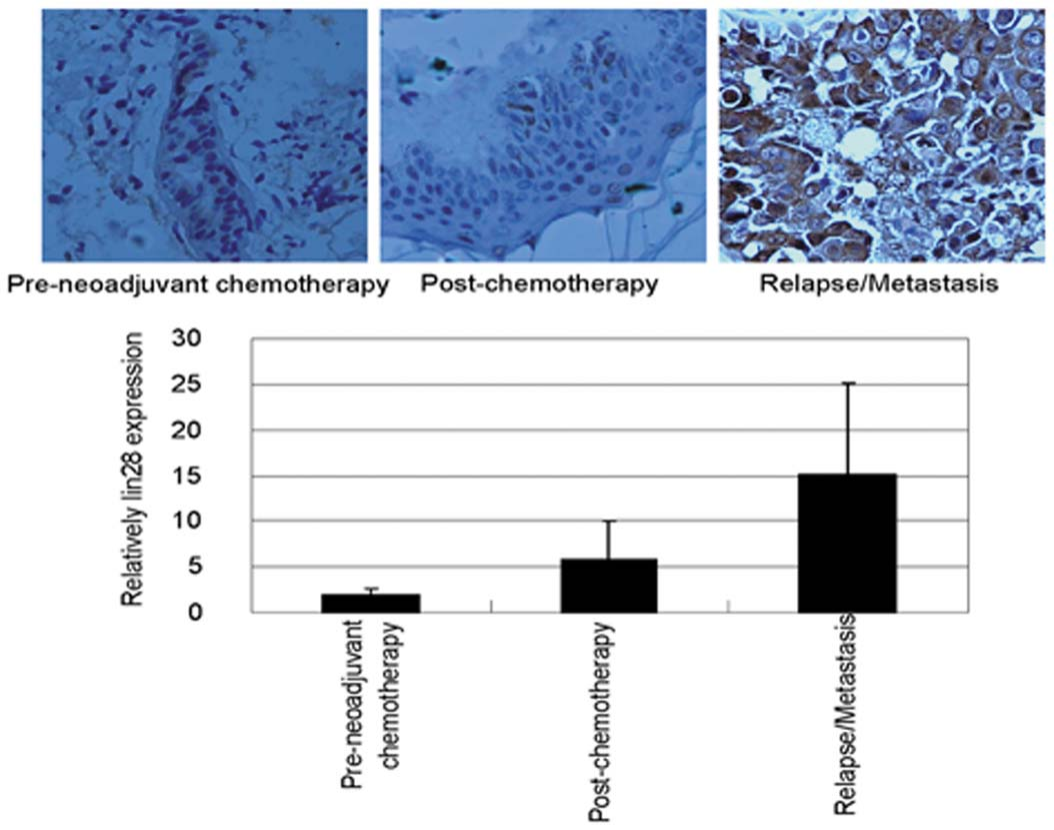
Lin28 is highly expressed in post-neoadjuvant and relapse or metastasis tumor tissues. The expression of Lin 28 in tumor tissues was determined by IHC.

To determine whether Lin28 is also associated with relapse or metastasis of breast cancer, we examined the expression of Lin28 in tumor tissues from various patients. Lin28 was measured in nine paired samples of breast cancer tissue including pre-neoadjuvant chemotherapy, post-neoadjuvant (operative), and relapse/metastasis. The results showed that after neoadjuvant chemotherapy, Lin28 expression was dramatically increased (p = 0.005) (Fig. 4). Moreover, Lin28 was dramatically upregulated in local recurrent or metastatic breast cancer tissues compared to primary tissues (p = 0.002) or operative tissues (p = 0.02) (Fig. 4). These results suggest that the breast cancer cells expressing low Lin28 levels were more sensitive and prone to die, but the breast cancer cells expressing high Lin28 levels were more resistant and able to survive. We also found that the post-neoadjuvant tissues had high Lin28 expression, and Lin28 expression was closely related with relapse or metastasis of breast cancer (Fig. 4).

### Lin28 Induces p21 and Rb and Inhibits Let-7 miRNA

**Figure 5:**
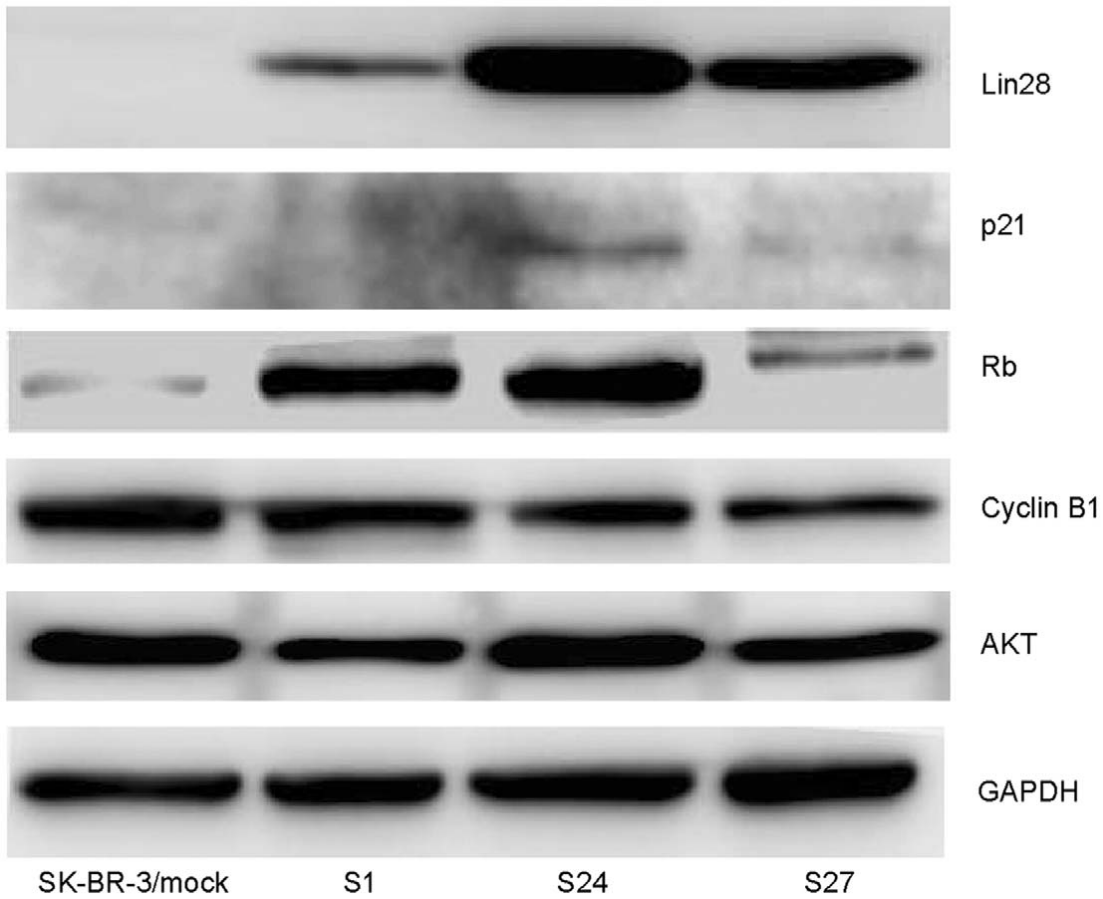
Lin28 regulates p21 and Rb expression. The expression of Lin28, p21, Rb, Cyclin B1 or Akt in SK-BR-3 cells stably expressing Lin28 was determined by Western blotting. The empty vector-transfected cells were used as controls. GAPDH was used as a loading control.

**Figure 6:**
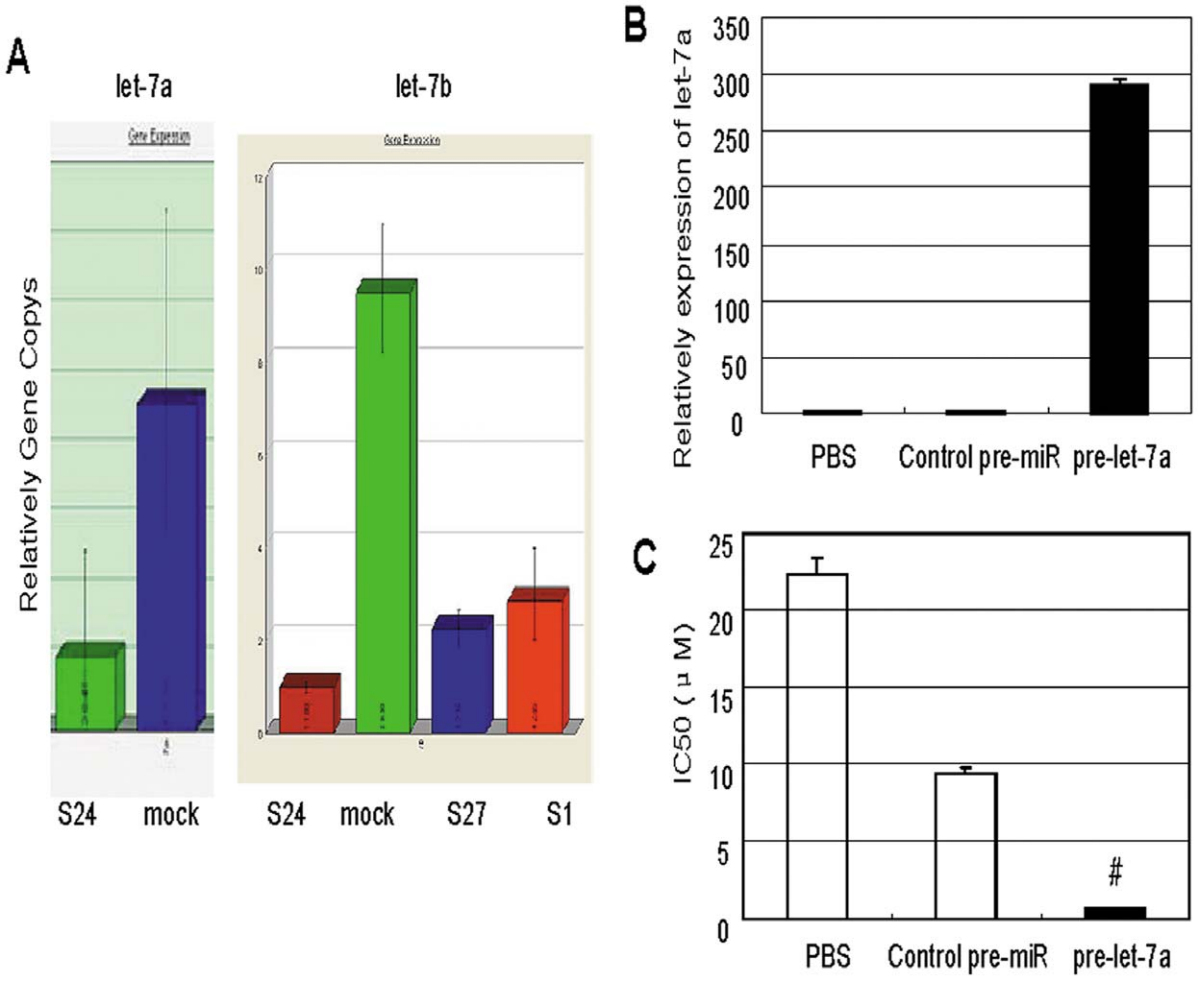
Lin28 downregulates Let-7 miRNA expression. (A). Expression of Let-7a or Let-7b miRNAs in SK-BR-3 cells stably expressing Lin28 was examined by qPCR. The empty vectortransfected cells were used as controls. (B). Cells were transfected with pre-Let-7a and expression of Let-7a was determined by qPCR in S1 cells stably expressing Lin28. (C). IC50 values for paclitaxel. S1 cells stably expressing Lin28 were transfected with pre-Let-7a, and cell viability and the IC50 of paclitaxel was determined by an MTT assay.

To further investigate the mechanism of Lin28-induced paclitaxel resistance, we measured the expression of some key genes and of Let-7 miRNA in the three SK-BR-3 clones that stably expressed Lin28. Western blotting analysis showed that p21 and Rb were significantly increased in the cancer cells stably expressing Lin28 compared to the mock controls, whereas Cyclin B1 and AKT expression did not change (Fig. 5). Real-time PCR analysis also showed that Let-7a and Let-7b miRNAs were dramatically decreased in the cancer cells stably expressing Lin28 compared to the mock controls (Fig. 6A). Transfection of cells with a Let-7 pre-miRNA significantly decreased the IC50 value of paclitaxel compared to that observed in the parental group or the pre-miRNA control group (p<0.01) (Fig. 6C).

## Discussion

In the present study, we investigated the association of Lin28 expression with chemoresistance in breast cancer cells. We found that Lin28 expression was upregulated in paclitaxel-resistant breast cancer cells and that Lin28 transfection induced paclitaxel resistance in breast cancer cells. We also showed that overexpression of Lin28 effectively induced p21 and Rb expression and inhibited let-7 miRNA. To our knowledge, this is the first finding that demonstrates that Lin28 expression is a possible mechanism of chemoresistance in breast cancer and suggests that Lin28 could be a potential target to overcome chemoresistance in breast cancer.

Chemotherapy is one of the major treatments for patients with cancers. However, chemoresistance remains a major factor that limits the effectiveness of chemotherapy. Therefore, identification of the tumor response to chemotherapy has long been a goal of oncologists and biologists. In the last century, scientists have observed an association between chemoresistance and the expression of several candidate genes, such as p53 [7], Ras [8], Raf-1 [9], Bcl-2 [10] and Survivin [11]. Recently, cancer stem cells have emerged as a contributor to chemoresistance through the preferential activation of the DNA damage checkpoint response and an increase in DNA repair capacity [12]. Furthermore, changes in some miRNA levels have been shown to be associated with chemotherapy sensitivity [13]; [14].

Previous studies have shown that Lin28 is overexpressed in hepatocellular carcinoma and that overexpression of this gene promotes cancer cell proliferation in vitro [4], but no reports have shown a relationship between the dysregulation of Lin28 and the chemoresistance of cancer cells. In this study, we explored the possible mechanisms of action of Lin28. We postulated that Lin28 is a potency factor of stem cells. Overexpression of Lin28 may give some “stemness” to cancer cells, thereby allowing them to acquire the ability of cancer stem cells to escape from chemotherapy. Lin28 has been shown to be associated with the processing of some miRNAs [15]; [16]. For example, Lin28 inhibited the processing of pri-let-7 in vitro and decreased endogenous levels of mature let-7 family members. All Let-7 family members tested were substantially upregulated upon knockdown of Lin28, whereas the levels of other miRNAs were unchanged. These data suggest that Lin28 has a preference for selectively blocking the processing of Let-7 family pri-miRNAs at the microprocessor step.

Let-7 has been reported to play a tumor suppressor role in lung and breast cancer by repressing oncogenes such as Hmga2 [17] and Ras [12]; [18], which suggests that disruption of Let-7 processing by activation of Lin28 could promote the oncogenic phenotype. A chemosensitive state can be created when the selected let-7 family of miRNA is overexpressed in vitro in ovarian cancer cells, whereas decreasing their levels caused chemoresistance [19]. Our current study showed that Let-7a and Let-7b miRNAs were significantly decreased in cancer cells stably expressing Lin28. Thus, Lin28 might change the chemosensitivity of breast cancer cells by affecting the miRNA processing.

## Materials and Methods

### Cell Culture and Chemicals

The human lung cancer cell lines (H460, H322), human colon cancer cell line (DLD1) and human breast cancer cell lines (SK-BR-3, MCF7, Bcap-7, MDA-231, T47D) were obtained from American Type Culture Collection (ATCC, Manassas, VA) and cultured in Dulbecco’s Modified Eagle’s Medium (DMEM) supplemented with 10% heat-inactivated fetal calf serum, 100 units/mL penicillin, and 100 mg/mL streptomycin (Invitrogen, Carlsbad, CA). In all of the experiments, cells were grown at 37°C in an atmosphere of 5% CO_2_. Paclitaxel was purchased from Sigma (St. Louis, MO) and dissolved in DMSO.

### Patients

For immunohistochemical staining, nine paired samples were obtained from breast cancer tissues routinely treated with neoadjuvant chemotherapy and operation and from local recurrent or metastatic tissue. Following the guidelines of the Ethics Committee, patients gave informed consent and samples were obtained at the Zhejiang University College of Medicine Department of Surgical Oncology at Sir Run Run Shaw Hospital from October 2002 to May 2010.

### miRNA Transfection

Cells were plated in six-well culture plates and transfected with 50 nM pre-let-7a purchased from Applied Biosystems (Carlsbad, CA) or control pre-miRNA according to the manufacturer’s protocol.

### Quantitative Real-time Polymerase Chain Reaction (PCR)

Real-time PCR was performed using the TaqMan MicroRNA Reverse Transcription Kit and the Fast Real-Time PCR System (Applied Biosystems, Carlsbad, CA) according to the manufacturer’s protocols. The fold change of let-7a microRNA levels was calculated and normalized to a hsa-mir-423 loading control.

### Immunohistochemical (IHC) Assay

IHC analysis for Lin28 expression was performed on formalin-fixed, paraffin-embedded sections of 9 paired samples obtained from breast cancer tissues pre-neoadjuvant chemotherapy and/or surgery and from local relapse or metastatic tissues. The slides were deparaffinized in xylene and rehydrated in gradient ethanol solutions. Endogenous peroxidase was blocked with 0.3% H_2_O_2_ in methanol for five minutes. The slides were immersed in 10 mM citric buffer (pH 6.0) with heat for 15 minutes for antigen retrieval. Nonspecific binding was blocked by preincubation with 10% fetal calf serum in PBS with 0.01% sodium azide, and the slides were incubated in a humid chamber for 1 hour with an antibody against Lin28 (rabbit polyclonal, Santa Cruz, 1∶80). After washing three times in PBS, the slides were incubated with the EnVision-HRP complex (undiluted, DAKO) for 60 minutes. The slides were visualized with diaminobenzidine (DAKO Corp, Houston, TX) and then counterstained with hematoxylin. For substitute negative controls, the primary antibody was replaced with phosphate-buffered saline. The expression of the antibodies was assessed semiquantitatively by estimating the strength and percentage of tumor cells with positive nuclei or cytoplasm staining on whole tumor slides. Scores were calculated based on the strength of the staining times the percentage of tumor cells with positive staining. All of the slides were examined and scored independently by two experienced pathologists to avoid subjective biases.

### Cell Viability Assay

Cell viability was measured using an MTT [3-(4,5-Dimethylthiazol-2-yl)-2,5-Diphenyltetrazolium Bromide] assay (Amresco, Solon, OH) according to the manufacturer’s protocol. Cells were seeded in 96-well plates at a density of 5×10^3^ cells per well. After treatment, the cells were incubated with 5 mg/ml MTT for 4 h. Then, the medium was removed and 150 µl of sterilized DMSO solution was added, followed by incubation at 37°C for 4 h. The absorbance of the reaction solution at 490 nm was measured, and these data were used to make growth curves. The cell growth inhibition rate was (1-absorbance of experimental group/absorbance of control group) ×100%.

### Western Blot Analysis

Cell lysates were separated by electrophoresis on a 4–15% sodium dodecyl sulphate-polyacrylamide gradient minigel (SDS-PAGE) (Bio-Rad, Hercules, CA) and electrophoretically transferred to a nitrocellulose membrane (Amersham Pharmacia, Piscataway, NJ). Western blots were probed with antibodies against Lin28, p21 (Santa Cruz Biotechnology, Santa Cruz, CA), AKT (Cell Signaling, Beverly, MA) or β-Actin (Sigma, St. Louis, MO). The protein bands were detected by enhanced chemiluminescence (Amersham Pharmacia, Piscataway, NJ).

### Flow Cytometric Assay

Cells (2×10^5^ per well) were plated in a six-well plate and treated with paclitaxel. After 72 h, cells were fixed in 70% ethanol and stained with propidium iodide (PI). The DNA content was analyzed with an Epics Profile II flow cytometer (Beckman Coulter, Fullerton, CA) with Multicycle software (Phoenix Flow Systems, San Diego, CA). All of the experiments were repeated at least twice. Moreover, PI was used in conjunction with Annexin V (BD Biosciences, San Jose, CA) to determine if cells were viable, apoptotic, or necrotic.

### Densitometric Analysis

Scion Image Software (Frederick, MD) was used to determine the density of protein or mRNA bands detected by Western blots or RT-PCR. The data are expressed as an arbitrary unit.

### Statistical Analysis

All of the experiments were performed three times with triplicate samples. Analysis of variance and Student’s t test were used to compare the values of the test and control samples. P<0.05 was considered to be statistically significant. Statistica 6.1 software was used for all statistical analyses. The significance was evaluated by the paired t test.
